# Effect of the Roasting Conditions on the Physicochemical, Quality and Sensory Attributes of Coffee-Like Powder and Brew from Defatted Palm Date Seeds

**DOI:** 10.3390/foods8020061

**Published:** 2019-02-06

**Authors:** Mohammad Fikry, Yus Aniza Yusof, Alhussein M. Al-Awaadh, Russly Abdul Rahman, Nyuk Ling Chin, Esraa Mousa, Lee Sin Chang

**Affiliations:** 1 Department of Process and Food Engineering, Faculty of Engineering, Universiti Putra Malaysia, 43400 Serdang, Selangor, Malaysia; chinnl@upm.edu.my; 2 Department of Biosystems and Agricultural Engineering, Faculty of Agriculture, Benha University,13736 Moshtohor, Toukh, Qalyoubia Governorate, Egypt; 3 Laboratory of Halal Services, Halal Products Research Institute, Universiti Putra Malaysia, 43400 Selangor, Malaysia; 4 Department of Agricultural Engineering, King Saud University, P.O. Box 2460, Riyadh 11451, Saudi Arabia; assiry@ksu.edu.sa; 5 Department of Food Technology, Faculty of Food Science & Technology, University Putra Malaysia, 43400 Serdang, Selangor, Malaysia; russly@upm.edu.my; 6 Department of special food and nutrition, Food Technology Research Institute, Agricultural research center, Giza, Egypt; esraa_am228@yahoo.com; 7 Department of Food Science, Faculty of Food Science & Technology, University Putra Malaysia, 43400 Serdang, Selangor, Malaysia; lixinc@hotmail.com

**Keywords:** roasting, palm date seeds, optimization, antioxidants, total phenolic content, coffee-like brew

## Abstract

Developing a bioactive brew is a novel track for revalorization of palm date byproducts. The effect of roasting temperature (160, 180 and 200 °C ) and roasting time (10, 20 and 30 min) on the hardness of the roasted date seeds, moisture content of the defatted roasted date seed powder (DRDSP), bulk density of the DRDSP, color parameters of DRDSP, quality attributes (extraction yield, pH and browning index), the chemical properties (antioxidants and total phenolic content) and the sensory attributes (color, aroma, taste and overall preference) of the brew prepared from DRDSP was studied. The physicochemical, quality, and sensory attributes were found to be significantly influenced by the roasting temperature and time. Additionally, the models proposed could satisfactorily describe the changes in the different properties during the roasting process. The optimum conditions of the roasting process obtained using the superimposed contour plot were 199.9 °C and 21.5 min. In the longer term, the results of this study would be beneficial for the manufacturers of the date seeds powder and brew.

## 1. Introduction

**Figure 1:**
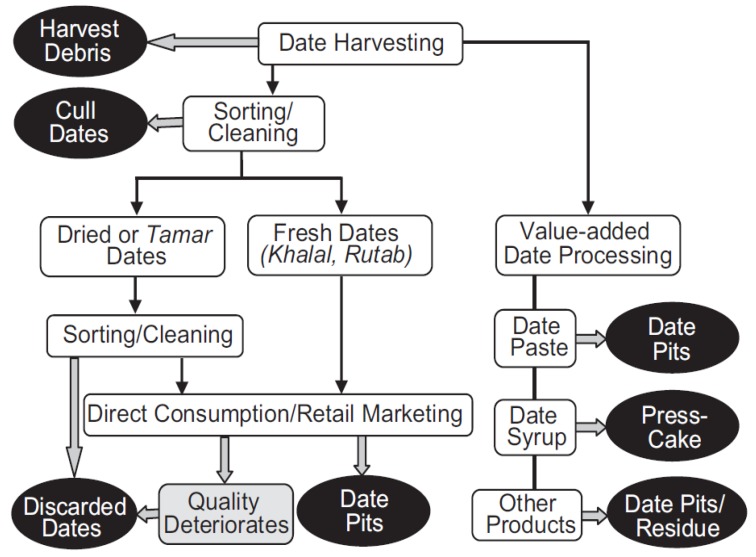
Palm date wastes resulting from different processes.

Palm dates (*Phoenix dactylifera* L.) are considered to be a nutritional component of the diet and a staple food source in most Middle Eastern and North African regions. Dates can be consumed either in a fresh form or as a derivative product, such as date-syrup, date-honey, date-jam, date-vinegar, and date-paste. In 2016, the global production of palm date fruits was approximately 8.5 million tons [1]. It was reported that the average percentage of the date seeds is approximately 10% of the total weight of the whole date fruit in the tamr “fully ripening” stage [2]. Date seeds are generated during direct consumption or from the date processing industries [3] (Figure 1). Presently, these byproducts are generally discarded leading to environmental problems, or instead, are utilized as animal fodder. The lack of uses for this by-product for human food constitutes a real economic loss since it is rich in dietary fiber, phenolic compounds and antioxidants, which can also be extracted and used as therapeutic components [4]. It has been reported that dietary fiber has important therapeutic implications for certain conditions, such as diabetes, hyperlipidaemia, and obesity, and may provide a protective effect against hypertension, coronary heart disease, high cholesterol, colorectal and prostate cancers, and intestinal disorders [5]. Additionally, antioxidants and phenolic compounds could protect against major chronic diseases, such as different types of cancer, cardiovascular disease, coronary heart disease, atherosclerosis and neurological problems, as well as aiding in treating renal stones, bronchial asthma, coughing, hyperactivity and poor memory, helping to reduce blood pressure, relaxing the intestinal and uterine musculature, growing body protein by reducing fat, normalizing blood sugar, and comforting the pancreas [6].

Due to the foregoing mentioned benefits, several potential uses of date seeds have been identified and reported in the literature. These included date seed powder as an ingredient in food products such as ground beef [7], bakery products [8,9], chocolate [10] and non-caffeinated drinks [11,12]. Virtually, roasted date seed powder is being used in the Arabian region, including the Kingdom of Saudi Arabia (KSA) and the United Arab Emirates (UAE), for preparation of an alternative brew to coffee [12,13] to avoid negative health impacts, such as raising blood pressure, panic attacks, hypertension, gout, insomnia, indigestion, infertility, and inhibition of collagen creation in the skin, as well as depression and anxiety symptoms resulting from the high content of caffeine in coffee (20–40%) [11]. It was reported that a brew made from roasted palm date seeds can be safely consumed and served to people who are sensitive to caffeine and prefer to enjoy the characteristic flavor and aroma of caffeine-free coffee without the adverse effects [14,15,16]. Interestingly, roasted palm date seeds have similar aromatic compounds (alcohols and aldehydes) that exist in Arabica coffee brews [17,18] and, importantly, are caffeine free [12,13]. 

The roasting process is considered one of the production stages of the date seed brew due to its advantages such as (i) increasing the efficiency of post-processing, (ii) improving food quality, and (iii) prolonging the shelf life of foods. Importantly, both temperature and time are considered the most important conditions of the roasting process. Mendes, et al. [19] stated that several factors might affect the required optimal roasting conditions, such as the required roasting level, the type of roaster, cultivar, the degree of maturity, and water content of the fresh product. The roasting degree can be observed by the color of the product, weight loss, and the taste and odor developed by the changes in chemical proximate [20]. Hence, the optimization of roasting conditions is important to produce an acceptable product. 

The physicochemical properties and sensory attributes of foods are noticeably changed during the roasting process. Many published works have investigated the effect of roasting conditions on the properties of several products, such as *Pistacia terebinthus* [21], maize [22], hazelnut [23], African breadfruit seed [24], robusta coffee [19,25], hulled sesame seeds [26] and arabica and robusta coffee beans [20]. In addition, the effect of roasting temperatures and time on the antioxidants, phenolic compounds, browning index, and some quality and sensory attributes of different brews, such as carob extract [27], corn kernels [28], a coffee-like beverage from maize kernels [22], coffee brew [29] and Polish hazelnuts [30] has been investigated. However, to the best of the authors` knowledge, there have not been any literature reviews for ascertaining the optimum roasting conditions for the production of a brew from defatted roasted date seed powder (DRDSP). Thus, the present work aimed to: (1) study the influence of roasting temperature and time on the physicochemical and sensory attributes of the powder and brew; (2) establish models to estimate the properties of both powder and brew; and (3) optimize the roasting conditions for preparing the brew.

## 2. Materials and Methods

### 2.1. Preparation and Brewing of the Defatted Roasted Date Seed Powder (DRDSP) 

**Figure 2:**
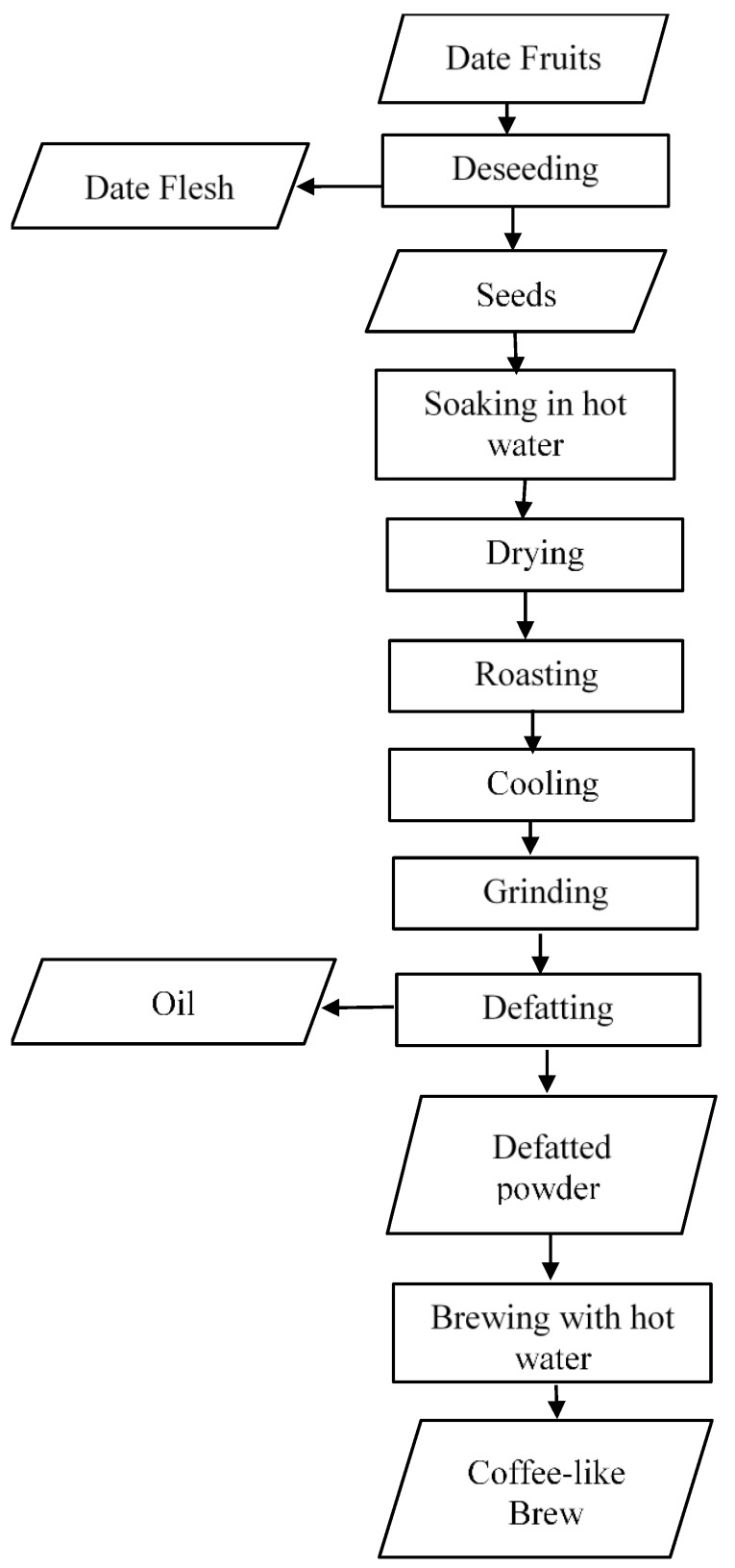
Flow chart of production of the brew from defatted roasted date seed powder.

According to the procedure used by Fikry, et al. [31], palm date fruits (Sukkari cultivar) were purchased from local Saudi markets, Riyadh, Saudi Arabia. The seeds were manually separated from the flesh and soaked in hot water at 100 °C for 1 h to remove any adhering residuals, then dried at 50 °C for 24 h to remove excess water from the surface of the seeds. The moisture content of the dried seeds was 3.4 ± 0.9% d.b. (dry base). Next, the date seeds were roasted by simulating the commercial roasting conditions of coffee using a natural convection oven (Memmert, UN, Schwabach, Germany) at three temperature levels of 160, 180 and 200 °C. Before beginning each roasting process, the oven was pre-heated for 1 h to ensure that the steady-state was reached. Then, around 100 ± 1.5 g of the date seed samples were placed on a piece of aluminum foil and spread as a thin layer on stainless steel trays in an oven. In order to obtain date seeds with different roasting degrees, each sample was removed from the oven at roasting time intervals of 10, 20 and 30 min. The samples were removed from the oven in less than 10 s so that steady-state conditions were maintained during sampling. All roasted date seeds were allowed to cool at room temperature and were then ground into powder using a hammer mill (Perten, 120, Finland) with a mesh size of 80 µm. The defatting process was performed according to the method used by Fikry, et al. [32]. Ten grams of ground roasted date seeds were used to extract the oil using a Soxhlet apparatus. The oil was extracted from date seed samples using an n-hexane solvent at 135 °C for 2 h. Next, the date seeds were placed in sealed polyethylene bags and stored in a refrigerator maintained at 7 ± 2 °C in order to avoid moisture absorption and to minimize any enzyme activity of the powder to avoid deterioration. The preparation processes of the DRDSP brew are portrayed in Figure 2. To obtain a good quality beverage, preliminary sensory testing was conducted by mixing the defatted roasted date seeds powder (DRDSP) and boiling distilled water at a ratio of 1:50 (*w/v*) and the mixture was put in a water bath at 80 °C for 1 h. Filtration of the extract through filter paper (No. 2, ADVANTEC, Tokyo, Japan) was conducted and the extracted beverage was used for investigating the quality indicators (extraction yield, browning index and pH), antioxidants and total phenolic contents. The extraction processes were triplicated, and the results are presented as mean ± standard deviation. 

### 2.2. Measurement of Hardness of the Roasted Seeds

The hardness of the roasted seeds was determined following the procedure used by Fikry, et al. [32]. Each single seed was placed horizontally between two flat parallel plates of an Instron Universal Testing 5566 Machine (Canton, MA, USA), then the sample was compressed using a 9.8 kg load cell at a crosshead speed of 10 mm/min. The hardness was measured using the maximum force in Newton at the first peak on the force-deformation curve. Ten measurements for each treatment were performed, and the results were reported as means and standard deviations. 

### 2.3. Measurement of Moisture Content of the DRDSP

According to the method described in Massini et al. [33], DRDSP samples (5 g) were dried at 100 °C in a convection oven (Memmert, UN, Germany) until achieving a constant weight. The moisture content (MC% d.b.) was calculated based on the final weight, and the results were summarized as the mean of triplicates.

### 2.4. Measurement of Bulk Density of the DRDSP

The bulk density ($\rho_{B})$ of the DRDSP samples was determined manually by pouring 2.5 g of the sample into a 10-mL graduated cylinder used for measuring the volume of the powder. The ratio of the mass of powder to the volume occupied by the DRDSP was used for calculation of the bulk density. 

### 2.5. Measurement of the Color of the DRDSP

The color attributes L* (darkness/lightness), a* (redness/greenness) and b* (blueness/yellowness) of the DRDSP samples were determined using a colorimeter (HunterLab, Color Quest® XE 3399, Reston, Virginia, USA). The instrument was standardized against white and black tiles before sample measurement. To determine the roasting degree, L*-value is a good choice for controlling the color changes during the roasting process, as the L*-value is likened to the color observation made by the operator. Table 1 reveals the roasting degree in relation to L*-values based on the classification described by Bolek and Ozdemir [21]. The color parameters were measured in triplicate and the results are presented as means and standard deviations. 

### 2.6. Determination of Extraction Yield and pH of the Brew

According to the method used by Youn and Chung [22], a sample of each of the extracted beverages (approximately 10 mL), was moved into a Petri dish and then dried at 105 °C in a convection oven (Memmert, UN, Schwabach, Germany) until the constant weight of the sample was reached. The extraction yield was calculated as a ratio of the weight of the extracted solids and the initial sample weight. The pH of the date seeds extract was measured by a Metrohm 654 pH meter with a glass electrode (Metrohm, Herisau, Switzerland).

### 2.7. Measurement of the Browning Index (BI) of the Brew

According to the procedure followed previously by Benjakul et al. [35], 50 µL of the date seed brew was diluted up to 2 mL with demineralized water. The browning index was measured by reading the absorbance of samples at 420 nm, after exactly 2 min in a 3-mL capacity cuvette (1 cm length) with a Lambda 25 UV-vis spectrophotometer (UV1601; Shimadzu, Kyoto, Japan). This index indicates the browned compounds resulting from caramelization and Maillard reactions, including melanoidins.

### 2.8. Analysis of Total Phenolic Content (TPC) 

According to the Folin–Ciocalteu procedure used by Singleton and Rossi [36], the total phenolic content of the hot water-extracted brew was determined. A sample of the brew (5 mL) and 5 mL Folin–Ciocalteu reagent were thoroughly mixed in a volumetric flask. After 3 min, 5 mL of 10% Na_2_CO_3_ solution was added, and the mixture was left for 1 h. The absorbance of the mixture was determined at 760 nm by using a spectrophotometer (UV1601; Shimadzu, Kyoto, Japan). The total concentration of phenolic compounds was determined by comparison with the absorbance of chlorogenic acid as standard. The total phenolic compounds were expressed as Gallic acid equivalent in mg/100 g dry weight (DW) date seed.

### 2.9. Determination of DPPH Radical Scavenging Activity 

DPPH radical scavenging activity of the DRDSP brews was determined according to the method previously used by Blois [37]. A sample of 0.2 mL of each brew was mixed with 0.8 mL of 0.4 mmol/L DPPH radical in ethanol. The mixture was vigorously shaken and left for 10 min. The absorbance of the mixture was determined at 525 nm with a spectrophotometer (UV1601; Shimadzu, Kyoto, Japan). The radical scavenging activity was calculated using Equation (1) as follows:

(1) $$\mathrm{Percentage}\;\mathrm{inhibition}=\;\left[1-\left(\frac{Abs_{\mathrm{sample}}}{Abs_{\mathrm{control}}}\right)\right]\times 100$$

### 2.10. Sensory Analysis of Palm Date Seeds Brew

Sensory evaluation was conducted with 30 assessors, consisting of Arabian people who are familiar with the natural drinks by considering the guidelines in the standard norm [38]. Ethically, assessors were informed and agreed to evaluate the brew before conducting the test, and were informed of the type of brew being assessed and then requested to evaluate four sensory attributes of the DRDSP brew samples (color, aroma, taste, and overall preference). The sensory assessment was applied in an environmentally controlled room (25 ± 2 °C) under white fluorescent light [21,22,29]. According to the method described by Mendes, et al. [19], a 9-point hedonic scale (1 = disliked extremely; 5 = neither liked nor disliked and 9 = liked extremely) was used by the assessors to determine how much they liked or disliked the samples. DRDSP brew samples were randomly evaluated in opaque, white and odorless cups, which were coded with random 3-digit numbers, and the samples were evaluated by all the assessors throughout 3 sessions. Assessors rinsed their mouths between samples using a glass of water. Data were presented as means and standard deviations. 

### 2.11. Experimental Design and Statistical Analysis

A 3-level 2-factor (3^2^) full factorial design was used as the experimental design as shown in Table 2. A 2-way ANOVA test was used to determine the effects of the independent variables on the dependent responses (hardness, moisture content, bulk density, extraction yield, pH, BI, DPPH radical scavenging activity and TPC). Multiple regression analysis was applied using MINITAB 18 (Minitab Inc, State College, PA, USA) to fit the proposed model including linear (*x*_1_, *x*_2_), quadratic (*x*_1_^2^, *x*_2_^2^) and interaction (*x*_1_*x*_2_) terms for the predictors (temperature, *x*_1_, and time, *x*_2_) to determine the regression coefficients and to draw the surface and contour plots. The significance levels of all the terms in the proposed equation were determined statistically by calculating the *F*-value at *p*-values of 0.01 or 0.05. Prasad and Nath [39] noted that an *R*^2^ value of at least 0.80 is satisfactory to describe the variability of the regression model and variance of the different properties. 

## 3. Results and Discussion

### 3.1. Effect of Roasting Conditions on the Physical Properties of DRDSP

Hardness, moisture content, density and color attributes are considered as quality criteria of DRDSP. Table 3 illustrates the means and standard deviations of hardness of the roasted seeds, moisture content of DRDSP, bulk density of DRDSP and color attributes (L*-value, a*-value and b*-value) of the DRDSP related to different roasting temperature and time.

Hardness is considered as an important indicator of the roasting level in date seeds. The decrease in the hardness of date seeds indicates that the force required to break the seeds decreases with the increase in the roasting temperature and time. In this case, the hardness of date seeds varied between 281 N and 2673.9 N at the different roasting temperature and time. The lowest hardness (281 N) was obtained at 200 °C for 30 min of roasting (Table 3). During roasting, it is suggested that the date seeds become more fragile as a result of decreasing moisture content and slackening of the structure resulting in the rise of its volume and porosity. Thus, dark roasted seeds require less grinding energy compared to medium roasted seeds. A similar trend has been detected for *Pistacia terebinthus* beans [21] and hazelnut [23]. 

Notably, the moisture content values of DRDSP generally decreased with increase in roasting temperature and time resulting from dehydration of the date seeds during the roasting process, as can be seen in Table 3. 

The bulk density of the DRDSP decreased as the roasting temperature and time increased (Table 3). The decrease of density could also be attributed to the rise of the volume. This could be due to the increase of porosity of the seeds’ structure, as determined by the rise in pressure of the internal gases (released CO_2_, water, and volatile substances) resulting from pyrolysis reactions [33]. Similar results have been reported for *Pistacia terebinthus* beans which were roasted at different roasting conditions using a fluidized bed roaster. 

**Figure 3:**
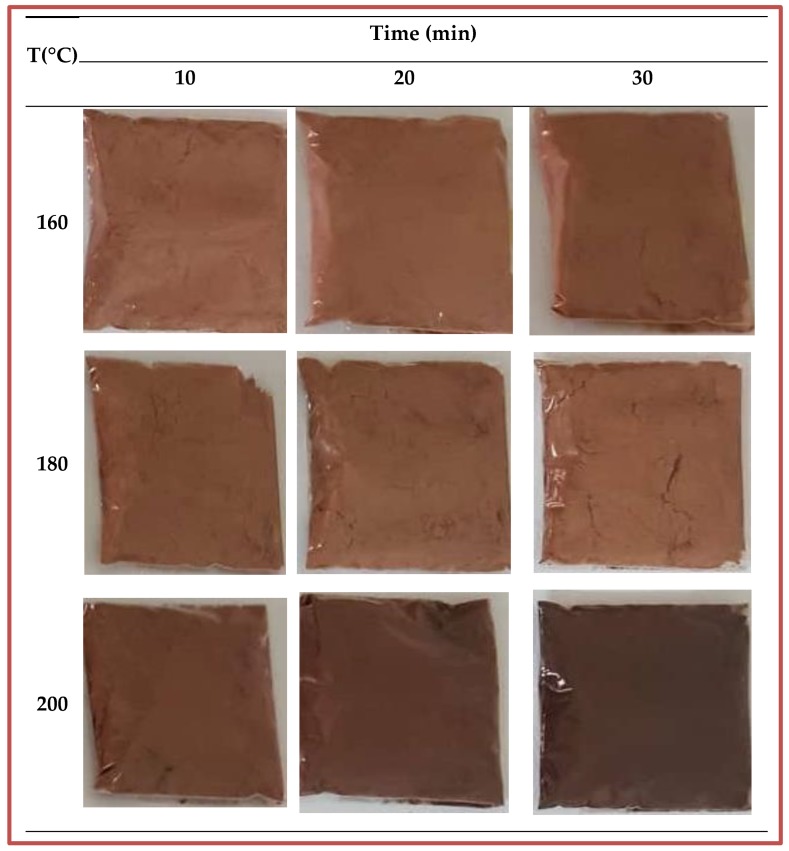
Color changes of the defatted roasted palm date seeds powder at different roasting temperature and times.

The color attributes of roasted products might also influence consumer acceptability. From Table 3, it can be observed that as the roasting temperature and time increase, L*-values decreased while a* and b*-values increased. Therefore, it can be suggested that the non-enzymatic browning and pyrolysis reactions occurring during the roasting process of DRDSP, which enhance the development of brown pigments, consequently give the DRDSP a darker color [33]. Furthermore, the reddish color of DRDSP increases as a*-value increases and the decrease in L*-value is an indication of its darkness. The roasting level of DRDSP ranges from “medium light” to “dark” based on the classification of L*-values mentioned in Table 1. These results are in agreement with those for *Pistacia terebinthus* beans [21], hazelnut [23], and coffee beans [20]. Color changes of the DRDSP at different roasting temperature and times can be depicted in Figure 3. 

### 3.2. Effect of Roasting Conditions on the Quality and Chemical Properties of the Brew

Extraction yield was defined as the mass of soluble solids in the extract. It can be seen in that the highest extraction yield of the brew was obtained with a roasting temperature of 200 °C and a roasting time of 30 min. Table 4 clearly shows that the extraction yield increased with the increase in the roasting temperature and time. These results can be explained by the softening of seeds’ texture for the material flux and the decomposition of insoluble polymers by the roasting temperature [40]. A similar trend was found for coffee -like maize brew [22]. 

Browning in roasted foods is mainly due to the development of non-enzymatic reactions such as the Maillard reaction and sugar caramelization [41]. Şahin, et, al. [27] stated that the Maillard reaction, which is a part of non-enzymatic browning reaction system, predominates when components such as reducing sugars and amines (amino acids, peptides or proteins) react with each other during thermal treatments in food processing. Thus, thermally processed foods generally contain various levels of Maillard reaction products, which are ideal time–temperature indicators for determining the extent of a thermal process. On the other hand, they also reported that Maillard reactions indicate some biological activities, both beneficial and harmful, such as antimicrobial, antioxidant, cytotoxic, cancerogenic or mutagenic activities, as well as reduction of allergenicity. 

The highest BI value of the brew was found at point of a roasting temperature of 200 °C and a roasting time of 30 min (Table 4). Also, depicts the increasing of BI with the increasing of the roasting temperature and time. This trend is like that reported for carob powder extract [27] and coffee brew [29]. 

Table 4 shows the variation in pH of the brew in relation to the roasting temperature and time. It was observed that the increase in roasting temperature and time caused a decrease of the pH of brew. It is noteworthy that the pH value of 5.67 significantly dropped to around 4.56 during the roasting period (Table 4). Similar findings for carob powder extracts were reported by Şahin, et, al. [27], and Yousif and Alghzawi [42]. It was suggested that the decrease in the pH value might be attributed to the formation of acidic caramelization by-products, such as pyruvic acid, and the formation of acidic Maillard products during the roasting process [42]. 

The antioxidative activity of the brews prepared from DRDSP under different roasting conditions was determined by investigating the DPPH radical scavenging activity. It can be seen from Table 4 that DPPH radical scavenging activity of the brew prepared from DRDSP increased with increasing roasting temperature and time. The DPPH radical scavenging activity values of the brew increased from 0.587% to 72.92% (Table 4). These findings are close to the results found for coffee brews [29], coffee-like maize beverage [22] and carob extract [27]. It was reported that the antioxidant activity of the different food materials increased as the roasting degree increases because of development of the Maillard reaction products during the roasting process [27]. To support this result, significant relationships between the DPPH and the BI values of the brews were detected (Table 5).

Phenolic compounds, commonly existing in food material, have many biological and functional properties that play a crucial role in food quality and human health [27]. The total phenolic content of the brew made from DRDSP increased with increase in roasting temperature and time (Table 4). Table 4 also indicates that the values of the total phenolic content of the brew varied between 7972.78 and 17191.67 mg/100 mg DW. It was suggested that the roasting process could cause evaporation of intracellular water, triggering chemical reactions that may change the lignocellulosic structure and promote protein denaturation, which may result in a greater availability of the phenolic compounds in the matrix [43]. Also, the increase in the TPC of the brews could be attributed to the formation of Maillard reactions with phenolic type structures, such as proanthocyanidins (condensed tannins) and gallic acid, during the roasting process [27]. It was previously reported that the formation of intermediate molecular weight melanoidins might be due to Maillard reactions and chlorogenic acid incorporation reactions between chlorogenic acids, sucrose, and amino acids/protein fragments during the initial roasting of coffee beans [44]. Moreover, Şahin, et, al. [27] stated that the TPC of the roasted products was higher than the TPC of the raw material and the preparation of roasted powders shows a large impact on the hydrolyzable tannins and gallic acid. The positive correlation between BI and TPC shown in Table 5 can support this explanation.

### 3.3. The Sensory Properties of the Brew as Affected by the Roasting Conditions

The sensory attributes of the brew made from the DRDSP under different roasting temperatures and times, namely, color, aroma, taste, and overall preference, were evaluated. A possible acceptability limit can be selected based on a score equal to 6.0 in a nine-point hedonic scale [45].

Color is a crucial indicator that can be used as a quality control indicator during roasting processes [19,41]. Table 6 reveals that the highest color score of the brew was 6.67 at a roasting temperature of 200 °C and a roasting time of 20 min. It can be clearly seen that the color score increased and then decreased as the roasting temperature and time increased. It was observed that the changes in accepting the color of the brew might be due to the increase of BI, which resulted from Maillard reactions [46]. Table 5 indicates that there is a positive relationship between BI and the sensory color of the brews.

The aroma is also regarded as an important quality indicator of date seeds brew. Table 6 indicates that the largest aroma scores of the brew were 6.60 for a roasting temperature of 200 °C and a roasting time of 10 min, meaning that the aroma of the brew is acceptable. Noticeably, the aroma scores increased and then decreased with increasing roasting temperature and time (Table 6). It was suggested that the changes in accepting the aroma might be due to the drop in the pH of the brews [47]. It can be observed from Table 5 that the correlation between the aroma and pH of the brews was found to be negative.

In terms of the taste attribute, it can be observed from Table 6 that the highest taste score of the brew was 7.33 obtained at roasting temperature 200 °C and roasting time 10 min. Clearly, taste scores increased and then decreased as the roasting temperature and time increased (Table 6). The changes in accepting the taste might be due to the decline in the pH of the brews that affects the acid flavor [47]. From Table 5, there is a negative correlation between the pH and the taste of the brews.

Table 6 indicates that the highest overall preference scores of the brew was 6.67 obtained at roasting temperature 200 °C and roasting time 20 min. In general, the overall preference scores of the brew increased and then decreased with the increase of roasting temperature and time. 

### 3.4. Data Validation 

**Figure 4:**
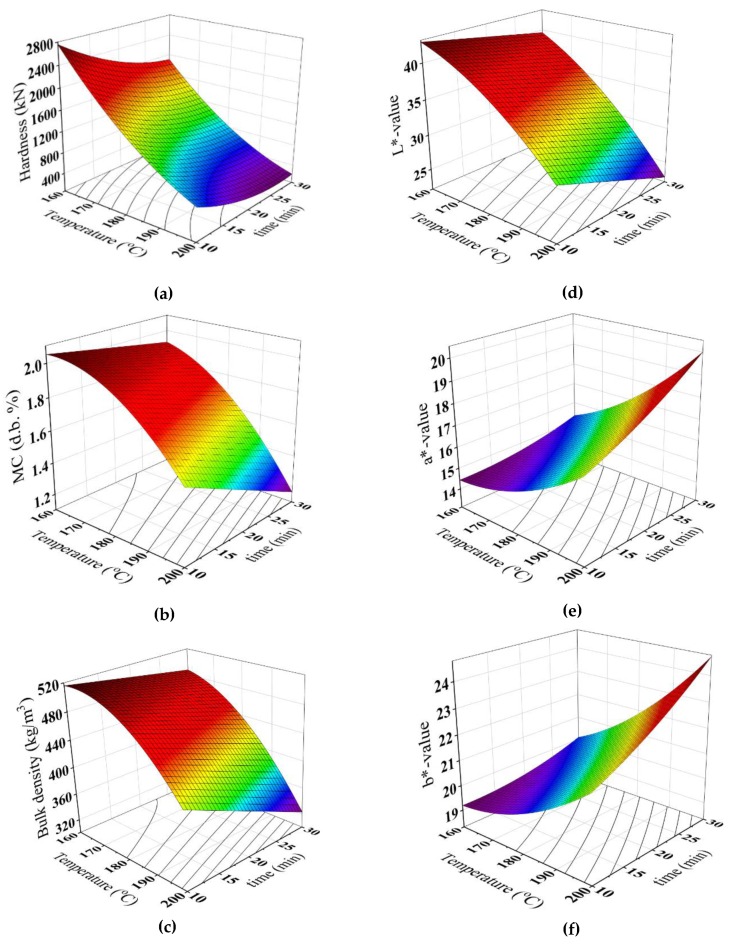
Response surface for (**a**) hardness (N) of whole seeds, (**b**) moisture content (MC d,b.%), (**c**) bulk density (kg/m^3^), (**d**) L*-value, (**e**) a*-value and (**f**) b*-value of the powder at different roasting conditions.

Regression analysis was applied to investigate the applicability of the second-degree polynomial model for describing the different properties of the whole seeds, powder and the brew. From Table 7, it can be seen that the hardness of the whole seeds, a*-value and b*-value were related to the linear, quadratic and interaction effects of roasting temperatures and time, while the moisture content, the bulk density and L*-value of the powder were found to be functions of the linear and quadratic effects of roasting temperatures, linearly related to time. The predicted data of the physical and color properties are outlined in Figure 4 using the predictive models.

**Figure 5:**
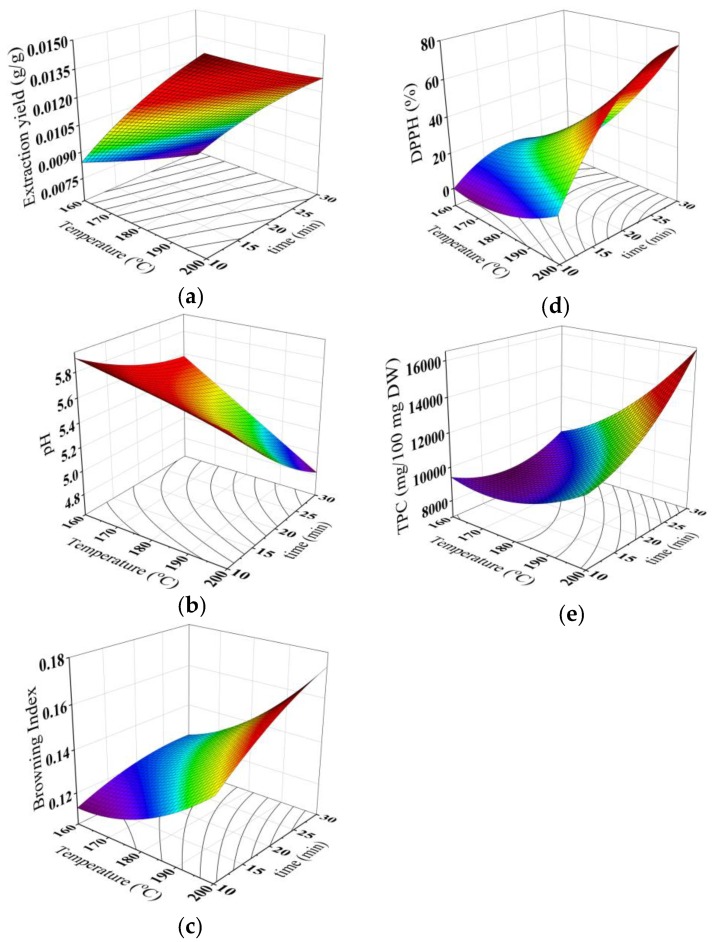
Response surface for (**a**) extraction yield, (**b**) pH, (**c**) browning index, (**d**) DPPH radical scavenging activity and (**e**) total phenolic contents of the brew at different roasting conditions.

Regards the quality properties of the brew, Table 7 reveals that the pH was linearly and quadratically related to the roasting time and related to the interaction between the temperature and time effect, whereas, the extraction yield was found to be a function of the linear and quadratic effects of the roasting temperature and also the quadratic effect of the roasting time. In addition, the BI values of the brew were found to be a function of the linear and quadratic effects of the roasting temperature and time. Table 7 also indicates that the DPPH radical scavenging activity and TPC of the brew were linearly and quadratically related to the roasting temperature and time, and revealed a significant effect of the interaction between the temperature and time. The extraction yield, pH, browning index, DPPH radical scavenging activity and total phenolic contents of the brew can be predicted using the model drawn in Figure 5.

**Figure 6:**
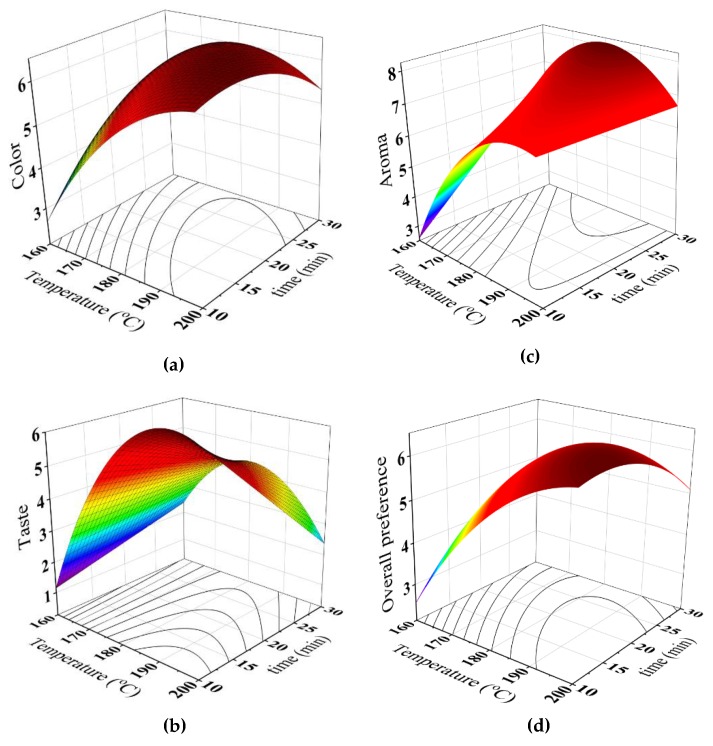
Response surface for (**a**) color, (**b**) taste, (**c**) aroma and (**d**) overall preference of the brew at different roasting conditions.

In terms of the sensory attributes, Table 7 shows that the color, aroma and overall preference scores of the brew were linearly and quadratically related to the roasting temperature and time. In addition, the interaction effect between the temperature and time was found to be significant. On the other hand, the taste score was found to be a function of the roasting temperature with linear and quadratic effects, and linearly related to the roasting time; the interaction between the roasting temperature and time was also found to be significant. Notably, Figure 6 was charted for predicting the sensory attributes as affected by the roasting temperature and time. It was suggested that the relationship between these responses and predictors (temperature and time) has been satisfactorily fitted to the model judging from *R*^2^ > 0.80 [39]. 

### 3.5. Determination of the Optimum Roasting Conditions 

To determine the optimal roasting conditions, superimposing the contour plots for the responses was used. Chambers and Wolf [48] stated that the consumer decision was affected by the overall preference of the products. Thus, in general, the overall preference scores can be used to determine the optimal point. In this case, an overall preference score of at least 6 (like) was selected as the minimum value for the preference. 

**Figure 7:**
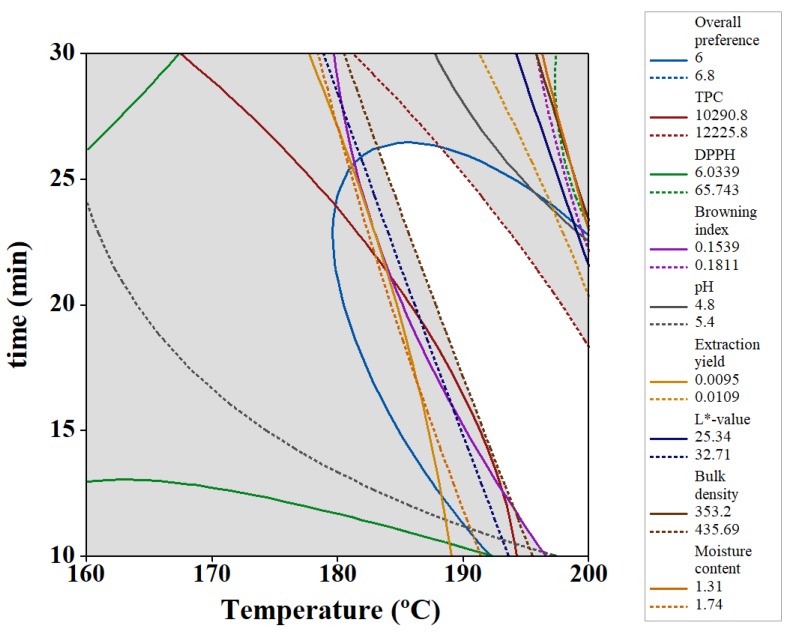
Superimposed contour plots of moisture content, bulk density, L*-value, pH, browning index, extraction yield, DPPH, TPC and overall preference of date seed brews as a function of roasting temperature and time.

To determine the optimal roasting point, boundaries of the overall impression score ≥ 6, 25.34 ≤ L* ≤ 32.71, 1.31% ≤ moisture content ≤ 1.74%, 353.2 kg/m^3^ ≤ bulk density ≤ 435.69 kg/m^3^, 4.8 ≤ pH ≤ 5.4, 0.1539 ≤ BI ≤ 0.1811, 0.0095% ≤ extraction yield ≤ 0.0109%, 6.0339 ≤ DPPH radical scavenging activity ≤ 65.743% and10,290.8 ≤ TPC ≤ 12,225.8 were considered. The optimum region (white shaded area) was generated by superimposing the boundaries; the contour plots of the above limitations can be seen in Figure 7. A brew corresponding to the above limitations would be prepared using the predicted optimum conditions of T = 199.99 °C and t = 21.5 min. 

## 4. Conclusion

A coffee-like brew prepared from roasted date seeds is considered as a bioactive drink that can be used as a therapeutic beverage. Hence, the present study describes for the first time changes in the hardness of roasted date seeds, the physical properties of DRDSP, and the quality and sensory attributes of DRDSP brew as affected by roasting conditions using full factorial experimental design. The results showed that the roasting temperature and time significantly affected the hardness of the roasted seeds, moisture content of DRDSP, bulk density of DRDSP, measured color attributes of DRDSP, extraction yield, pH, browning index, antioxidant activity, TPC, sensory color, aroma, taste and overall preference of the brews. A roasting temperature of 200 °C for 20 min resulted in a dark powder characterized by a moisture content of 1.33% d.b., a bulk density of 357.4 kg/m^3^, an L*-value of 25.36, an a*-value of 18.26, a b*-value of 22.95, an extraction yield of 0.01073 g/g, a pH of 4.90, a browning index of 0.181, DPPH radical scavenging activity of 64.05%, total phenolic content of 11719.72 mg/100 mg DW, and the highest sensory score. Models were developed for predicting the properties of the powder and brew as a function of the roasting temperature and time. Mutual correlations were observed between all chemical and sensory attributes of the date seed brew assessed in this work. The optimal roasting temperature and time were portrayed using contour plots in order to prepare date seed brews with high antioxidant activity and TPC, and good sensory attributes. As a result, the preferable characteristics of the brew from DRDSP could be obtained using the optimum roasting conditions (199.99 °C and 21.5 min). In the longer term, the present results could be beneficial for establishing analytical indicators to monitor the quality of date seeds during roasting and, thus, optimizing roasting conditions.

## Figures and Tables

**Table 1:** Roasting degree in relation to L*-value [34].

L*-Value	Roasting Index
≥57	Very light
42–56.99	Medium light
37–41.99	Medium
29–36.99	Medium dark
20.1–28.99	dark
≤20	Very dark

**Table 2:** Experimental design including process factors and their levels.

Treatments	Temperature, *x*_1_ (°C)		Time, *x*_2_ (min)	
	Coded	Actual	Coded	Actual
1	−1	160	−1	10
2	−1	160	0	20
3	−1	160	1	30
4	0	180	−1	10
5	0	180	0	20
6	0	180	1	30
7	1	200	−1	10
8	1	200	0	20
9	1	200	1	30

**Table 3:** Experimental data of physical and color properties of the defatted roasted date seed powder (DRDSP) as affected by the roasting conditions.

T (°C)	t (min)	Moisture Content (% d.b.)	Bulk Density (kg/m^3^)	Hardness (N)	L*-Value	a*-Value	b*-Value	Roasting Level
160	10	2.04 (0.01)	515.4 (4.20)	2673.93 (45.8)	42.70 (0.015)	14.39 (0.07)	19.16 (0.06)	Medium Light
	20	1.99 (0.02)	504 (4.2)	2226.3 (112)	41.33 (0.02)	14.63 (0.02)	19.39 (0.02)	Medium
	30	1.96 (0.02)	488.4 (4.2)	1941.6 (83.2)	38.42 (0.015)	15.35 (0.04)	20.12 (0.01)	Medium
180	10	1.90 (0.02)	493.01 (4.2)	1667.9 (74.3)	37.36 (0.02)	15.18 (0.02)	19.94 (0.04)	Medium
	20	1.84 (0.03)	475.66 (4.2)	901.8 (43.5)	35.26 (0.02)	15.82 (0.03)	20.58 (0.03)	Medium Dark
	30	1.69 (0.05)	431.49 (4.2)	756.6 (29.9)	32.67 (0.02)	17.03 (0.02)	21.79 (0.02)	Medium Dark
200	10	1.55 (0.02)	415.52 (4.2)	606.3 (11.8)	30.22 (0.015)	16.87 (0.03)	21.57 (0.04)	Medium Dark
	20	1.33 (0.02)	357.4 (4.2)	321.43 (68.6)	25.36 (0.01)	18.26 (0.03)	22.95 (0.05)	Dark
	30	1.21 (0.05)	330.09 (4.2)	281 (1.00)	22.03 (0.02)	20.02 (0.07)	24.70 (0.03)	Dark

d.b.: dry base; * Numbers in parentheses refer to the standard deviations of three replicates.

**Table 4:** Experimental data of quality and chemical properties of brew as affected by the roasting conditions.

T (°C)	t (min)	Extraction Yield (g/g)	pH	Browning Index (Abs at 420 nm)	DPPH Radical Scavenging Activity (%)	Total Phenolic Content (mg/100 mg DW)
160	10	0.0054 (0.0002) *	5.67 (0.06)	0.124 (0.0004)	0.587 (0.053)	7972.78 (383.2)
	20	0.0061 (0.0002)	5.46 (0.058)	0.132 (0.001)	2.165 (0.564)	8959.17 (751.1)
	30	0.0070 (0.0002)	5.37 (0.1)	0.135 (0.001)	4.811 (0.046)	9758.89 (589.2)
180	10	0.00853 (0.0002)	5.57 (0.06)	0.135 (0.001)	3.506 (0.123)	9760.56 (1375.9)
	20	0.00913 (0.0002)	5.16 (0.1)	0.145 (0.0004)	32.764 (4.569)	10,068.89 (317.9)
	30	0.00963 (0.0002)	4.93 (0.06)	0.155 (0.001)	18.539 (2.338)	11,029.17 (641.6)
200	10	0.01053 (0.0002)	5.37 (0.05)	0.158 (0.0003)	6.674 (0.554)	11,086.39 (498.5)
	20	0.01073 (0.0002)	4.90 (0.10)	0.181 (0.001)	64.046 (1.757)	11,719.72 (448.7)
	30	0.01163 (0.0002)	4.56 (0.06)	0.188 (0.001)	72.919 (0.250)	17,191.67 (1154)

* Numbers in parentheses refer to the standard deviations of three replicates. DW: dry weight.

**Table 5:** Pearson’s correlation matrix of the quality, chemical and sensory characteristics of the brew.

Property	pH	Antioxidants activity	Total phenolic contents	Browning Index	Color	Aroma	Taste	Overall Preference
pH	1							
Antioxidants activity	−0.823 **	1						
Total phenolic contents	−0.847 **	0.732 **	1					
Browning Index	−0.943 **	0.890 **	0.846 **	1				
Color	−0.585 **	0.465 *	0.278	0.592 **	1			
Aroma	−0.439 *	0.248	0.148	0.426 *	0.880 **	1		
Taste	−0.631 **	0.509 **	0.300	0.676 **	0.914 **	0.841 **	1	
Overall preference	−0.572 **	0.450 *	0.248	0.596 **	0.967 **	0.938 **	0.953 **	1

* and ** refer to the significance level *p* ≤ 0.05 and *p* ≤ 0.01, respectively.

**Table 6:** Experimental data of sensory attributes of the brew in relation to roasting temperature and time.

Roasting Conditions		Color	Aroma	Taste	Overall Preference
T (°C)	t (min)				
160	10	2.73 (0.46)	2.60 (0.51)	1.93 (0.42)	2.73 (0.46)
	20	4.33 (0.49)	3.53 (0.52)	2.40 (0.40)	4.07 (0.26)
	30	5.27 (0.46)	4.47 (0.52)	4.53 (0.31)	4.80 (0.41)
180	10	5.33 (0.49)	5.67 (0.49)	5.47 (0.31)	5.07 (0.26)
	20	5.93 (0.26)	6.00 (0.38)	6.67 (0.46)	5.67 (0.49)
	30	6.13 (0.52)	6.53 (0.52)	7.00 (0.20)	6.13 (0.35)
200	10	6.07 (0.59)	6.60 (0.51)	7.33 (0.23)	6.13 (0.35)
	20	6.67 (0.49)	6.20 (0.41)	5.53 (0.61)	6.67 (0.49)
	30	5.13 (0.52)	4.27 (0.88)	4.40 (0.40)	4.73 (0.46)

* Numbers in parentheses refer to the standard deviations of three replicates.

**Table 7:** Regression coefficients of the second order polynomial equation for the physical, color, quality and sensory attributes.

Property	Regression Coefficients of the Second-Degree Model						
	$y_{n}=\beta_{0}+\beta_{1}x_{1}+\beta_{2}x_{2}+\beta_{11}x_{1}{}^{2}+\beta_{22}x_{2}{}^{2}+\beta_{12}x_{1}x_{2}$						
	*β*_0_	*β*_1_	*β*_11_	*β*_2_	*β*_22_	*β*_12_	*R* ^2^
Hardness (N)	31489 ***	−266.8 ***	0.582 ***	−192.9 ***	1.714 ***	0.509 ***	0.985
Moisture content (% d.b.)	−7.31 ***	0.1133 ***	−0.00034 ***	0.0463 ***	0.000056	−0.000329 ***	0.989
Bulk Density (kg/m^3^)	−1690 ***	26.51 ***	−0.07896 ***	10.26 ***	−0.0004	−0.073 ***	0.989
L*-value	−52.7 **	1.312 ***	−0.00441 ***	0.625 ***	−0.00076	−0.004892 **	0.996
a*-value	55.05 ***	−0.482 ***	0.001436 ***	−0.4879 ***	0.002361 ***	0.002738 ***	0.999
b*-value	57.66 ***	−0.4568 ***	0.001363 ***	−0.4851 ***	0.0024 ***	0.002713 ***	0.999
Extraction yield (g/g)	−0.05376 **	0.000558 **	−0.000001 **	0.000106	0.000001 *	−0.000001 *	0.994
pH	6.27 **	−0.0014	0.0000	0.0569 **	0.000667 *	−0.000625 **	0.974
Browning index	0.6276 **	−0.006357 **	−0.002062 **	0.000019 **	−0.000034 **	0.000025 **	0.993
DPPH radical scavenging activity (%)	576 *	−6.65 *	0.01733 *	−6.47 *	−0.1515 **	0.0775 **	0.935
Total phenolic contents (mg/100 mg DW)	93199 *	−891 *	2.41 *	−1421 **	10.18 *	6.41 **	0.871
Sensory color	−82.78 **	0.8228 **	−0.001917 **	1.0333 **	−0.005333 **	−0.004333 **	0.936
Sensory aroma	−141.31 **	1.469 **	−0.003639 **	1.0406 **	−0.00222 *	−0.00525 **	0.915
Sensory taste	−194.5 **	2.028 **	−0.005056 **	1.167 **	0.00244	−0.006917 **	0.921
Overall preference	−83.33 **	0.8261 **	−0.001917 **	1.0222 **	−0.005333 **	−0.004333 **	0.928

*, ** and *** indicate that the effect is significant at *p*-value ≤ 0.05, *p*-value ≤ 0.01 and *p*-value ≤ 0.001, respectively.
